# Effectiveness and Cost-Effectiveness of an App-Based Mindfulness Breast Care Program for Reducing Body Image Distress and Stigma Among Survivors of Breast Cancer: Randomized Controlled Trial

**DOI:** 10.2196/85913

**Published:** 2026-04-24

**Authors:** Huicong Lin, Yujing Zhong, Shuang Zheng, Xiaoying Jiang, Yun Xu, Sally Chan, Shengjie Liu, Ka Ming Chow, Qingmo Yang, Wenhe Huang, Jiemin Zhu

**Affiliations:** 1 Department of Obstetrics and Gynecology, Women and Children's Hospital School of Medicine Xiamen University Xiamen China; 2 School of Medicine Xiamen University Xiamen China; 3 Department of Nuclear Medicine General Hospital of Central Theater Command Wuhan China; 4 School of Nursing Fujian Medical University Fuzhou China; 5 School of Social and Behavioral Sciences Nanjing University Nanjing China; 6 Center for Mental Health Education and Research Nanjing University Nanjing China; 7 President Office Tung Wah College Hong Kong China; 8 Department of Thyroid and Breast Surgery, Weifang People's Hospital Shandong Second Medical University Weifang China; 9 The Nethersole School of Nursing, Faculty of Medicine The Chinese University of Hong Kong Hong Kong China; 10 Department of Breast Surgery, The First Affiliated Hospital Xiamen University Xiamen China; 11 Cancer Centre and Department of Breast and Thyroid Surgery, Xiang’an Hospital Xiamen University Xiamen China; 12 Xiamen Research Centre of Clinical Medicine in Breast & Thyroid Cancers Xiamen University Xiamen China; 13 Department of Nursing, Women and Children's Hospital School of Medicine Xiamen University Xiamen China

**Keywords:** app-based mindfulness intervention, body image distress, breast cancer, cost-effectiveness, quality of life, randomized controlled trial, stigma

## Abstract

**Background:**

Breast cancer surgery and corresponding treatments have significant residual effects on survivors of breast cancer in China. Body image distress and stigma are persistent challenges that negatively affect their quality of life. Accessible, sustainable, and cost-effective support remains scarce.

**Objective:**

This study aimed to evaluate the effectiveness and cost-effectiveness of an app-based mindfulness breast care (MBC) program in addressing body image distress and stigma for survivors of breast cancer.

**Methods:**

We carried out a randomized controlled trial in 2 university-affiliated hospitals in China. Survivors of breast cancer who had completed primary treatments and had mobile phone internet access were recruited and randomly assigned at a 1:1 ratio to the intervention (3-month MBC program plus routine care) or the control group (routine care alone). Under the conceptualization of mindfulness-based cognitive therapy, the MBC program was developed, including three modules: (1) Library, (2) Mindfulness Yoga, and (3) Mindfulness Practices. The primary outcomes measured were body image distress and stigma, and secondary outcomes included sleep quality, social support, and quality of life (physical and mental well-being). Assessments were conducted at baseline, 3 months (T1), and 6 months (T2). Multiple imputation was used to handle missing data and generalized estimating equations were fitted to evaluate the effectiveness. The incremental cost per quality-adjusted life year (QALY) gained was used to measure cost-effectiveness.

**Results:**

A total of 192 survivors of breast cancer participated in the baseline assessment, with 155 completing the 2 follow-up surveys. The median total usage duration was 199.60 (IQR 70.90-451.31; mean 360.59, SD 511.72) minutes, and total login frequency was 39.50 (IQR 19.00-86.50; mean 57.02, SD 50.26) times. The reduction in body image distress at T2 (adjusted mean difference −1.91; 95% CI −3.40 to −0.42; *P*=.01; *d*=−0.31), the reduction in stigma at T1 (adjusted mean difference −5.83; 95% CI −8.46 to −3.20; *P*<.001; *d*=−0.61) and T2 (adjusted mean difference −7.79; 95% CI −10.62 to −4.97; *P*<.001; *d*=−0.82), and the improvement in mental well-being at T1 (adjusted mean difference 4.44; 95% CI 1.70 to 7.18; *P*=.002; *d*=0.43) were statistically significantly greater in the intervention group compared with the control group. No statistically significant group differences were observed regarding sleep quality, social support, and physical well-being. The cost-effectiveness analysis showed that the intervention group gained more QALYs than the control group at T2 (adjusted mean difference 0.008; 95% CI 0.004 to 0.016; *P*=.01). The incremental cost per QALY gained at T2 was US $19,431.25, indicating a 57% probability that the MBC program is a cost-effective intervention at a threshold of US $37,530, three times the 2023 gross domestic product per capita of China.

**Conclusions:**

An app-based MBC program was effective and potentially cost-effective and had the promise to be scalable for clinical practice.

**Trial Registration:**

Chinese Clinical Trial Registry ChiCTR2200059952; https://www.chictr.org.cn/showproj.html?proj=167247

## Introduction

With early detections and advancements in medical technology, the number of survivors of breast cancer has been continuously increasing [1]. However, breast cancer surgery and corresponding treatments have significant residual effects on survivors of breast cancer. The entire or partial loss of breasts after surgery and adjuvant therapy–induced menopause or vaginal dryness often significantly negatively impacts women’s feminine identity and body image, causing body image distress [2-4]. Body image distress is typically conceptualized as the negative mental representation of an individual’s body, as well as their perceived dissatisfied state of being themselves [5]. Previous studies show that many survivors of breast cancer experience body image distress [6], even many years after recovery [7]. Sustained body image distress often leads to low self-esteem, resulting in stigma [8].

Stigma is proposed as a deeply discreditable attribute that associates an individual with an undesirable stereotype [9]. More than 80% of survivors of breast cancer in China perceive stigma [10]. Cancer-related stigma may be culturally based, especially in traditional Chinese medicine, where breast cancer is connected with some unfavorable personality traits (eg, narrow-mindedness) [10]. Some survivors of breast cancer even believe that they are to blame for their disease because of their immoral behavior or misfortune [11]. Stigma often prevents survivors of breast cancer from accepting the kindness of others and participating in social activities [12], results in poor sleep quality [13], and ultimately adversely affects their quality of life (QoL) [14].

Various approaches have been explored to alleviate body image distress and stigma among survivors of breast cancer. Psychoeducational therapies and physical activities have been proven to be effective interventions for alleviating body image distress and stigma among survivors of breast cancer [15,16]. Recently, mindfulness-based cognitive therapy (MBCT) has also emerged as an increasingly popular approach for supporting survivors of breast cancer [17,18]. MBCT applied cognitive behavioral therapy skills to practice mindfulness-based stress reduction [19]. Cognitive behavioral therapy provides psychoeducation about the interactions between thoughts, emotions, and behaviors [20], while mindfulness-based stress reduction emphasizes enhancing mindfulness skills through regular meditation practices [21]. The combination of the 2 can contribute to better control of feelings, thoughts, and behavior and ultimately to achieve mind stability [19]. MBCT guided some group-based skills training programs, involving psychoeducation, yoga, and regular mindfulness practices, to help patients deal with chronic conditions [22,23]. These programs have been proven to alleviate patients’ body image distress for young patients with cancer [24], stigma for female patients with schizophrenia [25], and psychological distress for patients with cancer [26]. However, there remains a paucity of such programs specially targeting survivors of breast cancer to address their unique psychological challenges in relation to body image distress and stigma.

Further, traditional face-to-face programs delivering MBCT are time-intensive for patients and resource-intensive for health care providers [27]. With the advantage of a broad reach, the internet may provide a convenient and cost-effective platform. Several internet-based programs have been developed to offer MBCT [27,28], but few such programs include health economic evaluation [29]. Compen et al [30] demonstrated the potential cost-effectiveness of internet-based individual MBCT in alleviating psychological distress in patients with cancer. However, this internet-based program was delivered individually and recruited patients with various types of cancers [30]. The cost-effectiveness of internet-based group MBCT remains unknown for survivors of breast cancer.

In 2023, nearly 1.079 billion individuals in China could surf the internet through their mobile phones [31]. WeChat, the most popular free mobile social platform in China, offers an easily accessible channel to deliver support and service to survivors of breast cancer [32]. Under the conceptualization of MBCT, we developed an app-based mindfulness breast care (MBC) program on the WeChat platform. We hypothesized that the MBC program (lasting 3 months) plus routine care (the intervention group) would outperform routine care alone (the control group) in reducing body image distress and stigma, while enhancing sleep quality, social support, and QoL. Additionally, the MBC program would be cost-effective.

## Methods

### Study Design and Participants

We conducted a randomized controlled trial (RCT; Multimedia Appendices 1 and 2). The trial was registered with the Chinese Clinical Trial Registry (trial number ChiCTR2200059952; date of registration: May 14, 2022).

Eligible participants were women (1) with breast cancer who had completed primary treatments (surgical operation, chemotherapy, and/or radiotherapy) and were ready to be discharged, (2) who had mobile phone internet access, and (3) who were literate in Mandarin. Participants diagnosed with other concurrent cancers (such as ovarian cancer) or psychiatric disorders were excluded.

We recruited participants from 2 university-affiliated hospitals. One hospital is located in a central city and performs 400-600 breast surgeries each year. The other hospital is located in a rural area and has about 100 breast surgeries annually. Of these, approximately 50% of survivors of breast cancer met the aforementioned inclusion criteria. After primary treatments, survivors of breast cancer were often followed up every 3 months after discharge. There were no other app-based programs to support survivors of breast cancer at the 2 participating hospitals during the study period.

### Procedures

The breast surgeons introduced the MBC program to eligible women in the Breast Cancer Unit. If women showed interest, the researchers approached them, confirmed their eligibility, and explained the program in detail. Eligible participants signed the consent form, completed a paper-based baseline assessment (T0), and were randomly assigned to different groups.

Participants in the intervention group scanned the Quick Scan QR code, downloaded the app of the MBC program from the WeChat platform, and used their accounts (phone numbers) and passports (changeable later) to log in to the program. Afterwards, trained researchers provided approximately 20-minute onsite program training, including reading contents in the Library module, watching videos in the Mindfulness Yoga module, and participating in videoconferences in the Mindfulness Practices module. The usernames expired 3 months after activation.

All participants were invited to complete follow-up paper questionnaires at 3 months (T1) and 6 months (T2). These evaluation time points were chosen because most internet-based interventions documented the greatest benefits within 3 months [33], and some benefits might be endured at 6 months [34]. If some participants did not come back to their hospitals at either follow-up time point to complete the paper questionnaires, the researchers sent the online survey with the same questionnaires via the “Questionnaire Star” platform, which had a feature that only when all questions were answered, the online questionnaire was able to be submitted. If several scales were ticked with the same answers consecutively, the questionnaires were returned to participants to double-check or refill. Participants received an appreciation gift of approximately US $3 after each evaluation (direct delivery of a gift after completing the paper questionnaires and WeChat red packet after completing the online questionnaires).

### Randomization and Masking

Internet Research Randomizer [35], with selected block sizes of 4, 6, and 8, was used to generate the group allocation. Participants were randomly assigned at a 1:1 ratio to the intervention (MBC program plus routine care) or the control group (routine care alone) according to sequential participants’ recruitment. Neither the data collectors nor the participants were blinded to group allocation.

### MBC Program

A multidisciplinary health care team and a Chinese IT company developed the MBC program. The development of the MBC program has been published [36]. A previous similar study on an internet-based MBCT program indicated that a 3-month intervention period was sufficient to improve psychological outcomes [37]. Thus, a 3-month MBC program was designed.

**Figure 1:**
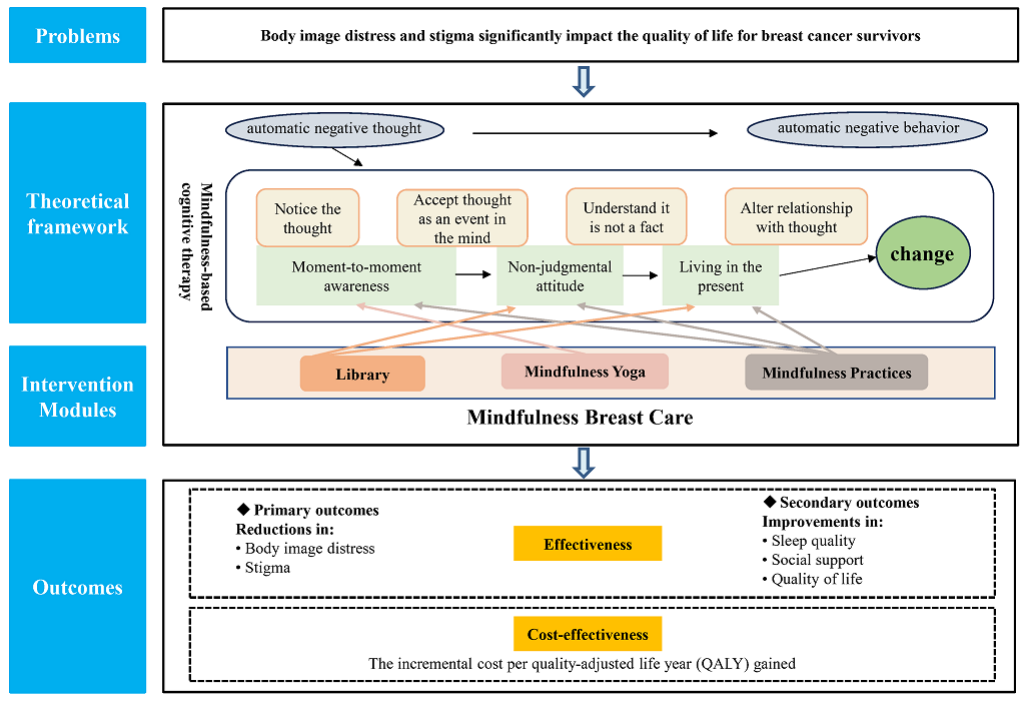
Framework of an app-based mindfulness breast care program for addressing body image distress and stigma.

The MBC program was based on MBCT. MBCT highlights the awareness of current feelings and thoughts, observes these with a nonjudgmental attitude, and ultimately develops individuals’ ability to achieve mind stability and live in the present (Figure 1) [19].

**Figure 2:**
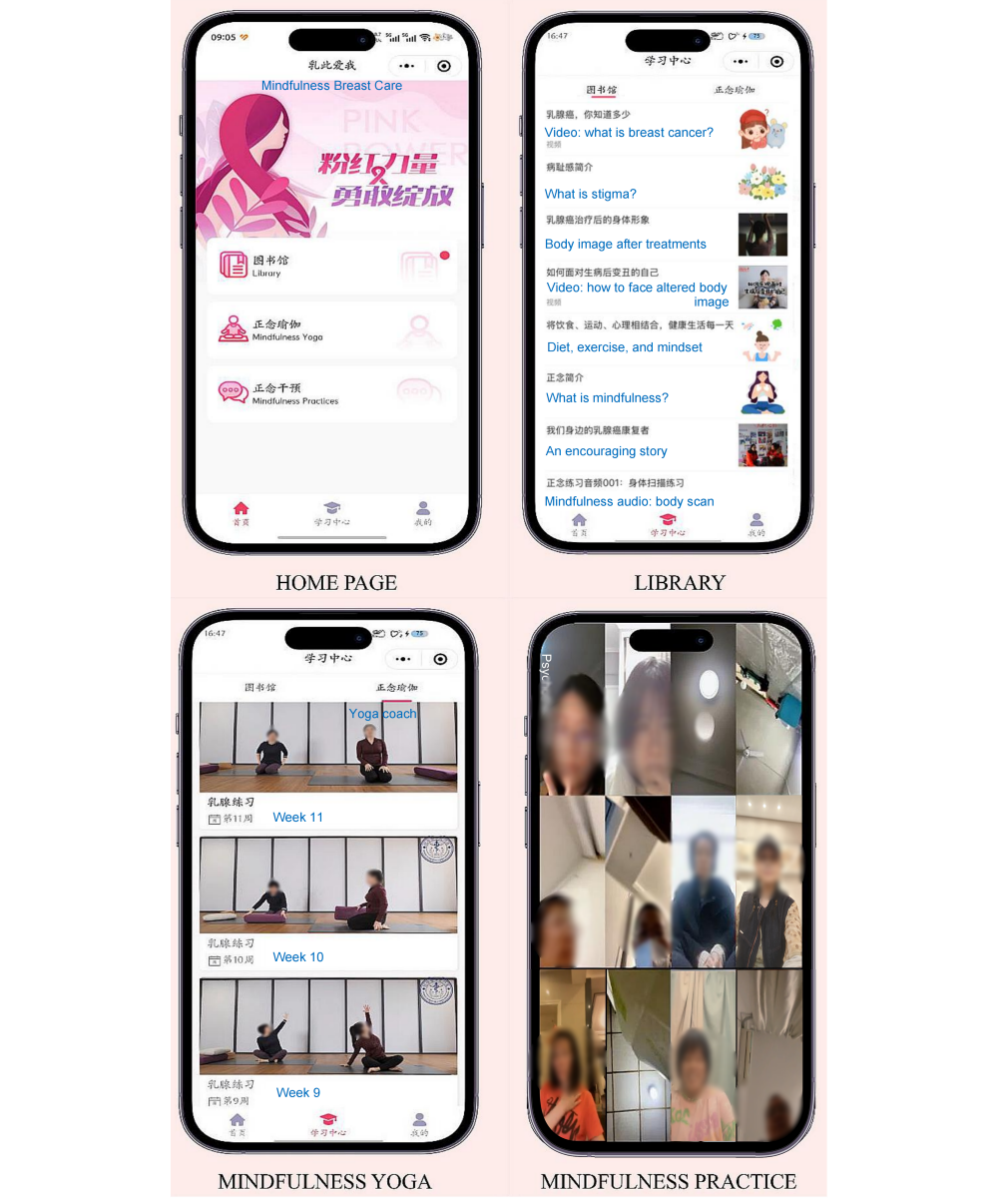
Screenshots of an app-based mindfulness breast care program.

This program included three modules: (1) Library, (2) Mindfulness Yoga, and (3) Mindfulness Practices (Figure 2). The Library module provided evidence-based educational information on breast cancer, the source of stigma, altered body image, strategies to manage stigma and body image distress, mindfulness, healthy lifestyle, and recovery [38-40].

The Mindfulness Yoga module consisted of 12 short videos of Hatha yoga, following the principles of MBCT, which incorporated mindful awareness into physical postures and breathing exercises [39]. During mindfulness yoga practice, women were guided to observe their bodies and be aware of their thoughts instead of judging their bodily sensations.

The Mindfulness Practices module was offered in 12 weekly 45-minute videoconferences. In a prior face-to-face MBCT intervention, participants were placed in groups of 8-12 participants and 1 certified mindfulness psychotherapist per subgroup [41]. Thus, according to the recruitment sequence, approximately 10 participants were allocated to a corresponding subgroup with 1 psychotherapist for better communication. Based on the Mindful Way Workbook [42], full communication between the 2 certified psychotherapists was conducted to guarantee standardized mindfulness practices for each group. Mindfulness practices involved three main components (1) awareness-guided meditation to focus on bodily and breathing sensations, such as sitting meditation; (2) connection to the present moment by observing somatic sensations and movements through practices like a guided body scan; and (3) development of a nonjudgmental attitude by observing mental activity, including emotions, impulses, and thought patterns, through practices such as 2 ways of knowing.

Various strategies were adopted to enhance the MBC program fidelity, including keeping the intervention consistent and enhancing participant engagement. To keep intervention consistency, the researchers ensured the standard delivery of mindfulness practice through full communication between 2 psychotherapists. To increase participants’ engagement, the content in the Library and Mindfulness Yoga modules was updated each week, with an instant WeChat messaging reminder of the incoming new information and a new Yoga video sent to the participants.

The quality of the MBC program was assessed using the Mobile App Rating Scale (user version) among 5 survivors of breast cancer for pilot feedback [43], with an overall mean score of 3.96 out of 5, indicating that the program is generally credible, enjoyable, usable, and highly relevant to survivors of breast cancer’s medical conditions.

### Routine Care

Participants in 2 groups received routine care. Before discharge, nurses offer oral and written instructions on routine information after cancer treatment and follow-up education, including exercises to prevent upper limb lymphedema, infection control, nutrition, healthy lifestyle, and information on medical follow-up. At every follow-up visit, oncologists conduct medical assessments and adjust medications as needed. Nurses give general advice on monitoring and managing symptoms post treatment.

### Measures

#### Sample Characteristic Measures

A self-designed questionnaire was applied to collect participants’ baseline sociodemographic and clinical characteristics.

#### Primary Outcome Measures

The primary outcomes included body image distress and stigma. Body image distress was evaluated using the 10-item Body Image Scale (BIS) [44]. A higher total score on the BIS (range 0-30) indicates a greater level of body image distress. The Chinese version of the BIS has good reliability in individuals with breast cancer [45]. The baseline Cronbach α was 0.94 in this study.

Stigma was evaluated using the 24-item Social Impact Scale (SIS) [9]. A higher total score on the SIS (range 24-96) indicates a greater perceived stigma. The Chinese version of the SIS has good reliability in individuals with breast cancer [10]. The baseline Cronbach α was 0.85 in this study.

#### Secondary Outcome Measures

The secondary outcomes included sleep quality, social support, and QoL, which were evaluated using the Pittsburgh Sleep Quality Index (PSQI) [46], Multidimensional Scale of Perceived Social Support (MSPSS) [47], and the 36-item Short-Form Health Survey (SF-36) [48]. The SF-36 is summarized into 2 component scores: the Physical Component Summary (PCS) and the Mental Component Summary (MCS). Higher total scores on the aforementioned questionnaires (PSQI range 0-21; MSPSS range 12-84; and SF-36 PCS/MCS range 0-100) indicate worse sleep quality, better social support, and better physical and mental well-being, respectively. These questionnaires have been validated in Chinese individuals with breast cancer [49-51]. In this study, the baseline Cronbach α values of PSQI, MSPSS, and SF-36 were 0.74, 0.94, and 0.91, respectively.

#### Economic Evaluation Measures

The costs were considered from a societal perspective [52], including (1) fixed costs of the development of the app-based MBC program; (2) direct medical costs; and (3) indirect costs, namely income and time lost due to medical treatment [53]. Furthermore, the scores of the SF-36 were converted into a health utility value based on the Short-Form Six-Dimension (SF-6D) to calculate the quality-adjusted life years (QALYs) [54].

#### Usage Data

The background thread of the MBC program tracked and recorded 3-month usage data, which were measured as login frequency (number of times a participant logged in the program) and usage duration (total time from login to logout) of the entire program and each module. If one participant’s mobile phone was in standby mode or one participant was browsing other websites together, the MBC program ran in the background operational mode and it stopped calculating the participant’s usage data. Based on a prior web-based intervention for survivors of breast cancer [55], the minimal login frequency and minimal usage duration of expected use were defined as 10 times and 50 minutes during the whole course of the MBC program.

### Sample Size

The primary outcomes of body image distress and stigma were used to estimate effect size. Previous literature reported an effect size of 0.28 for body image distress [56] and 0.36 for stigma among patients with cancer [57]. Thus, the effect size of 0.28 was chosen to calculate the sample size. Using G*Power (version 3.1.9.7), a total of 154 participants (77 each group) were needed for 90% power at a significance level of .05 (2-sided). Accounting for a 20% dropout rate, a target sample size of 192 (96 per group) was determined.

### Statistical Analyses

#### Overview

Statistical analysis was performed using SPSS (version 26; IBM Corp) and R (version 4.3.3; R Foundation for Statistical Computing). Exploration of the missing-data structure indicated that the missingness was consistent with a missing-at-random mechanism. Therefore, to ensure robustness of parameter estimation, multiple imputation was conducted to handle missing data. Baseline comparisons were performed using the chi-square test or Fisher exact test for categorical variables, as well as independent-samples 2-sided *t* tests for continuous variables.

#### Effectiveness

Generalized estimating equations were applied to analyze repeated measurements at baseline, 3 months, and 6 months with group, time, and the group × time interaction as fixed effects. Demographic and clinical characteristics that significantly differed between groups (endocrine therapy) and baseline outcomes were adjusted during data analysis. An exchangeable working correlation structure was specified to account for within-subject correlation scores across repeated assessments, and robust standard errors were used to obtain valid inference even if the correlation structure was not specified. *P* values were adjusted with the Bonferroni correction to reduce the risk of inflated type I error. The adjusted estimated mean differences, 95% CIs, significance levels, and adjusted effect sizes were reported. The adjusted effect size (Cohen *d*) was calculated as the adjusted mean difference divided by the pooled unadjusted SD at baseline [58]. The minimally important clinical differences of the primary outcomes were evaluated as one-half the SD of the baseline score [59] using the distribution-based method [60]. To examine the robustness of the results regarding missing values for the primary outcomes, a sensitivity analysis was conducted with Markov chain Monte Carlo simulation imputation.

#### Cost-Effectiveness

An economic evaluation was performed to explore the cost-effectiveness of the MBC program in terms of incremental cost per QALY gained [61]. QALYs gained were calculated from health utility scores [54] using the area-under-the-curve method [62]. Total costs were calculated as the sum of the following three types of costs: (1) fixed costs associated with the app development, (2) direct medical costs, and (3) indirect costs.

Fixed costs included (1) costs for app development and maintenance, (2) costs of video production, and (3) consultation fees charged by mindfulness therapists (approximately US $44.78 per hour). According to the CHEERS (Consolidated Health Economic Evaluation Reporting Standards) statement (Multimedia Appendix 2) [52], the app was assumed to remain operational for 5 years. Fixed costs were amortized using a 3% discount rate with the following calculation formula [63] and then converted into 6-month average costs.



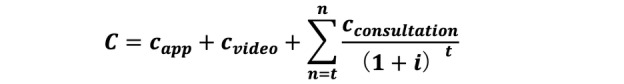



Consistent with standard trial-based economic evaluation methods [64], direct costs were extracted from medical records strictly within the predefined 6‑month study for each participant. Indirect costs were calculated as the average labor income multiplied by the time spent in the hospital from T0 to T2, including inpatient and outpatient days (times of clinic visits × 0.5 day per visit) [65] following the human capital approach [66]. According to 2023 statistics, the per capita annual income in Xiamen city was US $10,606.27, yielding a daily average income of US $40.80 [67]. All costs were converted to 2023 US dollars using the mean exchange rate averaged across 2022 and 2023 provided by the Bank of China foreign exchange rate.

Generalized estimating equations were fitted to evaluate the direct and indirect costs (adjusted for endocrine therapy) and the QALYs gained (adjusted for endocrine therapy and baseline health utility scores). Total costs and QALYs gained were used to estimate the incremental cost-effectiveness ratio (ICER) and net monetary benefit. According to the recommendation of the World Health Organization [68], 3 times the 2023 gross domestic product (GDP) per capita (US $12,510) was applied as the threshold [69]. The interpretation was as follows: if the ICER is less than 3 times the GDP (US $37,530), it is deemed cost-effective [68].

Sensitivity analysis was used to determine how the ICER was influenced by altering specific assumptions or parameters. The 2-tailed *t* tests used a bias-corrected and accelerated nonparametric bootstrapping method with 10,000 replications to estimate the 95% CI of incremental costs and QALYs at T2 [70]. The cost-effectiveness plane was graphically represented to illustrate the uncertainty surrounding the ICER at the threshold. Cost-effectiveness acceptability curves were used to display the net monetary benefits of the MBC program at various willingness-to-pay thresholds, ranging from US $0 to US $200,000 per QALY gained [71].

#### Usage Data and Its Relationships With Measurement Outcomes

Due to the highly skewed nature, the usage duration (minutes) and login frequency of the program were presented as median, mean, and maximum values. Spearman rank correlation was used to calculate the associations between MBC usage data and measurement outcomes at baseline, T1, and T2.

### Ethical Considerations

The trial was conducted in accordance with the Declaration of Helsinki. This study received approval from the ethics committees of First Affiliated Hospital, Xiamen University (approval number FA-2022-30) and Xiang’an Hospital, Xiamen University (approval number XAHLL2022027). All participants were assured of voluntary participation and provided written informed consent. All collected data regarding participants were confidential and anonymous. Participants received compensation (approximately US $3) each time they returned their questionnaires. The patients provided written informed consent to allow their image to be published.

## Results

### Sample Characteristics

**Figure 3:**
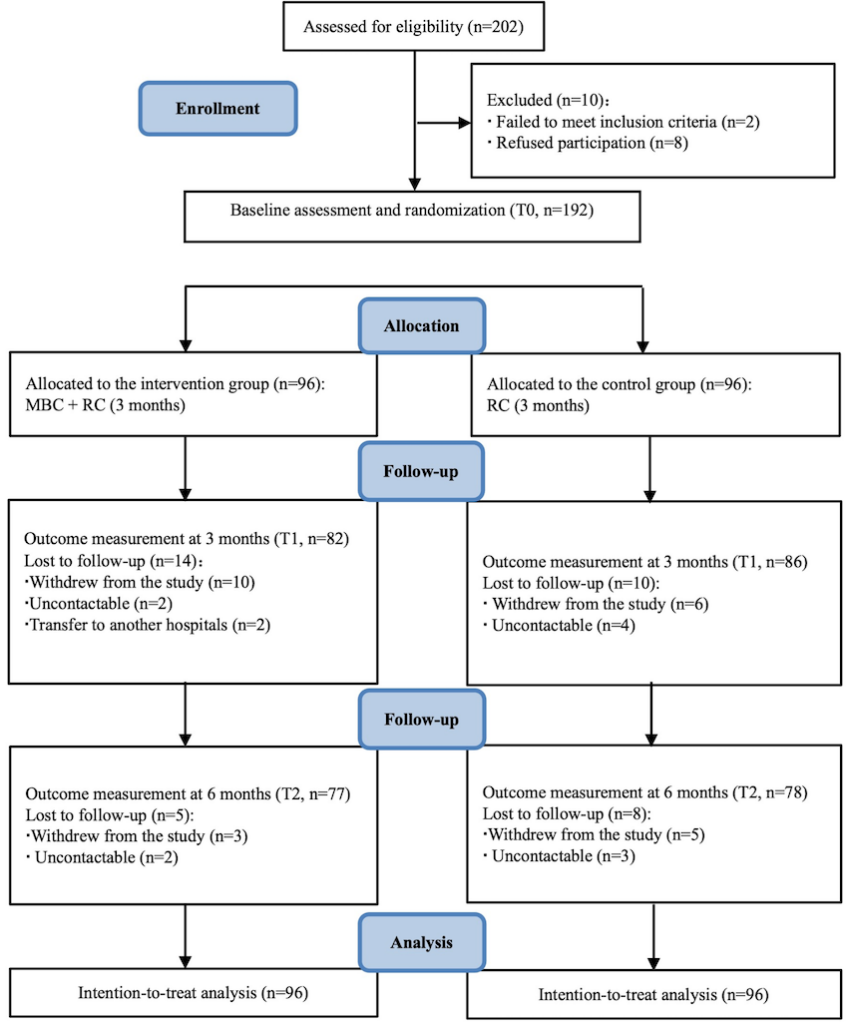
Trial profile (CONSORT flow diagram). CONSORT: Consolidated Standards of Reporting Trials; MBC: mindfulness breast care; RC: routine care.

We approached a total of 202 participants for eligibility assessment, and 192 (95.2%) completed baseline assessments. A total of 155 participants completed all follow-up assessments at T1 and T2, yielding a response rate of 80.7% (155/192; Figure 3). The participants (mean age 45.59, SD 8.74 years) were primarily diagnosed with stage II breast cancer (104/192, 54.2%). Most participants had an education at the senior high school level or above (114/192, 59.4%). Almost all participants (187/192, 97.4%) were diagnosed with breast cancer within 2 years. All participants (192/192, 100%) had surgical treatment and chemotherapy. Most participants underwent axillary lymph node dissection (153/192, 79.7%), endocrine therapy (114/192, 59.4%), targeted therapy (104/192, 54.2%), and radiotherapy (103/192, 53.6%). No group differences were found in baseline characteristics except for endocrine therapy (*P*=.04; Table 1).

**Table 1:** Participants’ characteristics and baseline health outcomes.

Variables			Overall (n=192)		Intervention (n=96)		Control (n=96)
Age (years), mean (SD)			45.59 (8.74)		44.64 (8.92)		46.55 (8.49)
**Marital status, n (%)**							
	Married or cohabitation	175 (91.1)		88 (91.7)		87 (90.6)	
	Separated or divorced or single	17 (8.9)		8 (8.3)		9 (9.4)	
**Education, n (%)**							
	Junior high school or lower	78 (40.6)		37 (38.5)		41 (42.7)	
	Senior high school or higher	114 (59.4)		59 (61.5)		55 (57.3)	
**Current employment, n (%)**							
	Employed	101 (52.6)		54 (56.3)		47 (49)	
	Unemployed or retired	91 (47.4)		42 (43.8)		49 (51)	
**Monthly family income (US $), n (%)**							
	<1492.54	144 (75)		73 (76)		71 (74)	
	≥1492.54	48 (25)		23 (24)		25 (26)	
**Payment methods, n (%)**							
	Medical insurance	179 (93.2)		87 (90.6)		179 (93.2)	
	Out of pocket money	13 (6.8)		9 (9.4)		13 (6.8)	
**Stage of breast cancer, n (%)**							
	I	46 (24)		24 (25)		22 (22.9)	
	II	104 (54.2)		55 (57.3)		49 (51)	
	III	42 (21.9)		17 (17.7)		25 (26)	
**Time form diagnosis, n (%)**							
	T ≤ 2 years	186 (97.4)		94 (97.9)		93 (96.9)	
	2< T ≤ 5 years	5 (2.6)		2 (2.1)		3 (3.1)	
**Surgery, n (%)**							
	Yes	192 (100)		96 (100)		96 (100)	
	No	0 (0)		0 (0)		0 (0)	
**Surgical methods, n (%)**							
	Breast-conserving surgery	40 (20.8)		19 (19.8)		21 (21.9)	
	Mastectomy	83 (43.2)		41 (42.7)		42 (43.8)	
	Mastectomy + breast reconstruction	69 (35.9)		36 (37.5)		33 (34.4)	
**Axillary lymph node dissection, n (%)**							
	Yes	153 (79.7)		76 (79.2)		77 (80.2)	
	No	39 (20.3)		20 (20.8)		19 (19.8)	
**Chemotherapy, n (%)**							
	Yes	192 (100)		96 (100)		96 (100)	
	No	0 (0)		0 (0)		0 (0)	
**Radiotherapy, n (%)**							
	Yes	103 (53.6)		49 (51.0)		54 (56.3)	
	No	89 (46.4)		47 (49.0)		42 (43.8)	
**Endocrine therapy, n (%)**							
	Yes	114 (59.4)		50 (52.1)		64 (66.7)	
	No	78 (40.6)		46 (47.9)		32 (33.3)	
**Targeted therapy, n (%)**							
	Yes	104 (54.2)		54 (56.3)		50 (52.1)	
	No	88 (45.8)		42 (43.8)		46 (47.9)	
**Traditional Chinese medicine treatment, n (%)**							
	Yes	35 (18.2)		21 (21.9)		14 (14.6)	
	No	157 (81.8)		75 (78.1)		82 (85.4)	
**Health outcomes,** **mean (SD)**							
	Body image distress (BIS^a^ scores)	6.71 (6.09)		6.76 (5.83)		6.67 (6.37)	
	Stigma (SIS^b^ scores)	52.58 (9.50)		52.88 (9.09)		52.29 (9.93)	
	Sleep quality (PSQI^c^ scores)	7.54 (3.88)		7.65 (3.69)		7.43 (4.08)	
	Social support (MSPSS^d^ scores)	66.04 (9.34)		65.88 (8.54)		66.21 (10.12)	
	Physical well-being (SF-36^e^ PCS^f^ scores)	36.01 (10.38)		36.54 (9.12)		35.47 (11.53)	
	Mental well-being (SF-36 MCS^g^ scores)	40.76 (10.37)		40.94 (10.27)		40.57 (10.51)	

^a^BIS: Body Image Scale.

^b^SIS: Social Impact Scale.

^c^PSQI: the Pittsburgh Sleep Quality Index.

^d^MSPSS: Multidimensional Scale of Perceived Social Support.

^e^SF-36: 36-item Short-Form Health Survey.

^f^PCS: the Physical Component Summary.

^g^MCS: the Mental Component Summary.

### Effectiveness

The intervention group showed a statistically significantly decrease in body image distress at T2 (adjusted mean difference, −1.91; 95% CI, −3.40 to −0.42; *P*=.01, *d*=−0.31), decrease in stigma at T1 (adjusted mean difference, −5.83; 95% CI, −8.46 to −3.20; *P*<.001, *d*=−0.61) and T2 (adjusted mean difference, −7.79; 95% CI, −10.62 to −4.97; *P*<.001, *d*=−0.82), and improvement in mental well-being at T1 (adjusted mean difference, 4.44; 95% CI, 1.70 to 7.18; *P*=.002, *d*=0.43) when compared to the control group (Table 2). No statistically significant group differences were observed regarding sleep quality, social support, and physical well-being. In our trial, only the between-group adjusted mean difference in stigma at T1 and T2 exceeded the minimally important clinical differences of stigma (the one-half SD of the baseline score). Sensitivity analysis yielded similar findings regarding the primary outcomes, which confirmed the robustness of the results (Table 3).

**Table 2:** Effects of mindfulness breast care program on health outcomes (N=192).

Outcomes^a^			Intervention group mean (SE)		Control group mean (SE)	Adjusted mean difference (95% CI)		*P* value^b^		Cohen *d* ^c^			
**Primary outcomes**													
**Body image distress (BIS scores)^d,e^**													
	T1	6.33 (0.52)		7.32 (0.52)			−0.99 (−2.43 to 0.46)		.18		−0.16		
	T2	6.32 (0.45)		8.23 (0.60)			−1.91 (−3.40 to −0.42)		.01		−0.31		
**Stigma (SIS scores)^e,f^**													
	T1	50.50 (0.99)		56.33 (1.01)			−5.83 (−8.46 to −3.20)		<.001		−0.61		
	T2	48.13 (0.99)		55.92 (1.01)			−7.79 (−10.62 to −4.97)		<.001		−0.82		
**Secondary outcomes**													
	**Sleep Quality (PSQI scores)^e,g^**												
	T1	7.09 (0.33)		7.29 (0.35)			−0.20 (−1.14 to 0.73)		.67		−0.05		
	T2	7.11 (0.34)		7.03 (0.34)			0.07 (−0.86 to 1.02)		.87		0.02		
**Social support (MSPSS scores)^h,i^**													
	T1	64.32 (1.14)		65.76 (1.12)			−1.44 (−4.63 to 1.74)		.37		−0.15		
	T2	64.72 (1.03)		61.91 (1.04)			2.71 (−0.15 to 5.58)		.06		0.29		
**Physical well-being (SF-36 PCS scores)^i,j,k^**													
	T1	39.69 (0.90)		38.11 (0.98)			1.58 (−1.03 to 4.19)		.24		0.15		
	T2	41.74 (0.95)		39.72 (0.92)			2.02 (−0.60 to 4.64)		.13		0.19		
**Mental well-being (SF-36 MCS scores)^i,l^**													
	T1	46.23 (1.02)		41.79 (0.95)			4.44 (1.70 to 7.18)		.002		0.43		
	T2	44.36 (0.96)		42.22 (0.94)			2.14 (−0.49 to 4.77)		.11		0.21		

^a^The intention-to-treat analysis was conducted using the multiple imputation method. Adjusted for endocrine therapy and baseline outcomes, generalized estimating equations were used to examine the effectiveness of the MBC program. Pairwise comparisons were conducted with Bonferroni adjustment.

^b^All *P* values for pairwise comparisons were Bonferroni-adjusted.

^c^Cohen *d* was calculated by dividing the adjusted mean difference by the pooled unadjusted standard deviation at baseline [58].

^d^BIS: Body Image Scale.

^e^An increase implies worsening and a decrease implies improvement.

^f^SIS: Social Impact Scale.

^g^PSQI: the Pittsburgh Sleep Quality Index.

^h^MSPSS: Multidimensional Scale of Perceived Social Support.

^i^An increase implies improvement, and a decrease implies worsening.

^j^SF-36: 36-item Short-Form Health Survey.

^k^PCS: the Physical Component Summary.

^l^MCS: the Mental Component Summary.

**Table 3:** Sensitivity analysis of primary outcomes.

Primary outcomes^a^		Intervention group mean (SE)	Control group mean (SE)	Adjusted mean difference (95% CI)	*P* value^b^	Cohen *d* ^c^
**Body image distress (BIS scores)^d,e^**						
	T1	4.41 (1.10)	5.54 (1.10)	−1.13 (−2.74 to 0.46)	.10	−0.19
	T2	4.62 (1.14)	6.27 (1.16)	−1.65 (−2.97 to −0.18)	.02	−0.27
**Stigma (SIS scores)^d,f^**						
	T1	47.02 (2.10)	52.80 (2.14)	−5.78 (−8.79 to −3.26)	<.001	−0.61
	T2	44.99 (2.14)	50.61 (2.18)	−5.62 (−8.18 to −3.20)	<.001	−0.59

^a^The intention-to-treat analysis was conducted using the Markov chain Monte Carlo simulation imputation. Generalized estimating equations were used to examine the effectiveness of the MBC program on primary outcomes. Pairwise comparisons were conducted with Bonferroni adjustment.

^b^All *P* values for pairwise comparisons were Bonferroni-adjusted.

^c^Cohen *d* was calculated by dividing the adjusted mean difference by the pooled unadjusted standard deviation at baseline [58].

^d^An increase implies worsening and a decrease implies improvement.

^e^BIS: Body Image Scale.

^f^SIS: Social Impact Scale.

**Figure 4:**
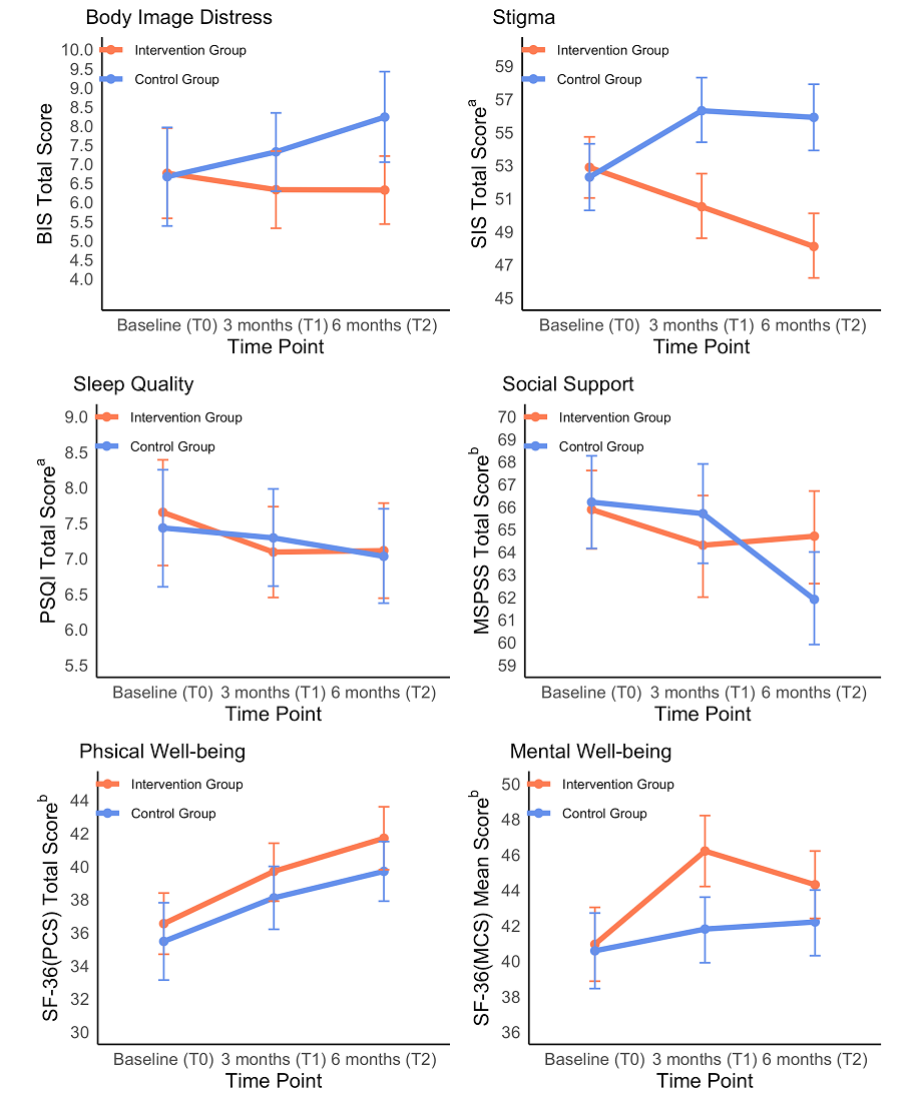
Adjusted mean values of the primary and secondary outcomes between two groups from T0 to T2. SIS: Social Impact Scale; BIS: Body Image Scale; PSQI: Pittsburgh Sleep Quality Index; MSPSS: Multidimensional Scale of Perceived Social Support; SF-36: Short Form-36 items; PCS: Physical Component Summary; MCS: Mental Component Summary. a An increase implies worsening and a decrease implies improvement. b An increase implies improvement and a decrease implies worsening.

At T1, a statistically significant time effect was found in stigma (T1:=−2.39; *P*=.001). At T2, statistically significant time effects were detected in social support (=−5.83; *P*=.004). Statistically significant time group interaction effects were identified for stigma at T1 and T2 (Group T1: =−5.89; *P*=.009; Group T2: =−6.64; *P*=.005) (Figure 4).

### Cost-Effectiveness

Fixed costs primarily comprised the following: (1) app costs totaling US $15,014.92, (2) video production amounting to US $746.27, and (3) first-year consultation fees charged by mindfulness therapists equaling US $4298.51. The total fixed cost over 5 years was US $36,037.68, with US $37.54 per participant for six months. There is no difference in adjusted direct medical costs and indirect costs between two groups at T2. The total costs were estimated at US $9295.45 for the intervention group and US $9140.00 for the control group with no statistically significant group difference (*P*=.92). The intervention group gained more QALYs than the control group at T2 (adjusted mean difference 0.008, 95% CI 0.004 to 0.016; *P*=.01). The incremental cost per QALY gained at T2 was US $19,431.25, making our program cost-effective at a threshold of three times of China’s 2023 GDP per capita (US $37,530; Table 4).

**Table 4:** Cost-effectiveness analysis for cost, health utility score, and quality adjusted life years gained by study time-point.

Variables^a^		Intervention group mean (SE)	Control group mean (SE)	Adjusted mean difference (95% CI)	*P* value
**Cost (US $), baseline to 6 months**					
	Fixed costs	37.54	0	—^b^	—
	Direct medical costs	9113.58 (1034.33)	9002.69 (1022.39)	111.04 (−2803.43 to 3025.37)	.94
	Indirect costs	144.33 (8.43)	137.31 (8.30)	7.03 (−16.10 to 30.15)	.55
	Total costs for 6 months	9295.45	9140.00	155.45 (−2773.28 to 3084.48)	.92
**SF-6D^b^ health utility score**					
	SF-6D value at T0	0.663 (0.014)	0.663 (0.014)	0.0001 (−0.019 to 0.019)	.997
	SF-6D value at T1	0.685 (0.014)	0.667 (0.014)	0.018 (−0.004 to 0.039)	.10
	SF-6D value at T2	0.681 (0.014)	0.674 (0.014)	0.007 (−0.013 to 0.021)	.06
**QALYs^c^ gained, baseline to 6 months**					
	QALYs (T0 to T1)	0.189 (0.002)	0.187 (0.002)	0.002 (–0.003 to 0.006)	.47
	QALYs (T0 to T2)	0.377 (0.003)	0.369 (0.003)	0.008 (0.004 to 0.016)	.01

^a^The data were analyzed using generalized estimating equations. The scores of the SF-36 were converted into a health utility value based on the SF-6D to calculate QALYs [54]. Costs were adjusted for endocrine therapy. The QALYs gained were adjusted for endocrine therapy and the SF-6D utility score at T0.

^b^Not applicable.

^c^SF-6D: Short Form Six-Dimension.

^d^QALYs: quality adjusted life years.

**Figure 5:**
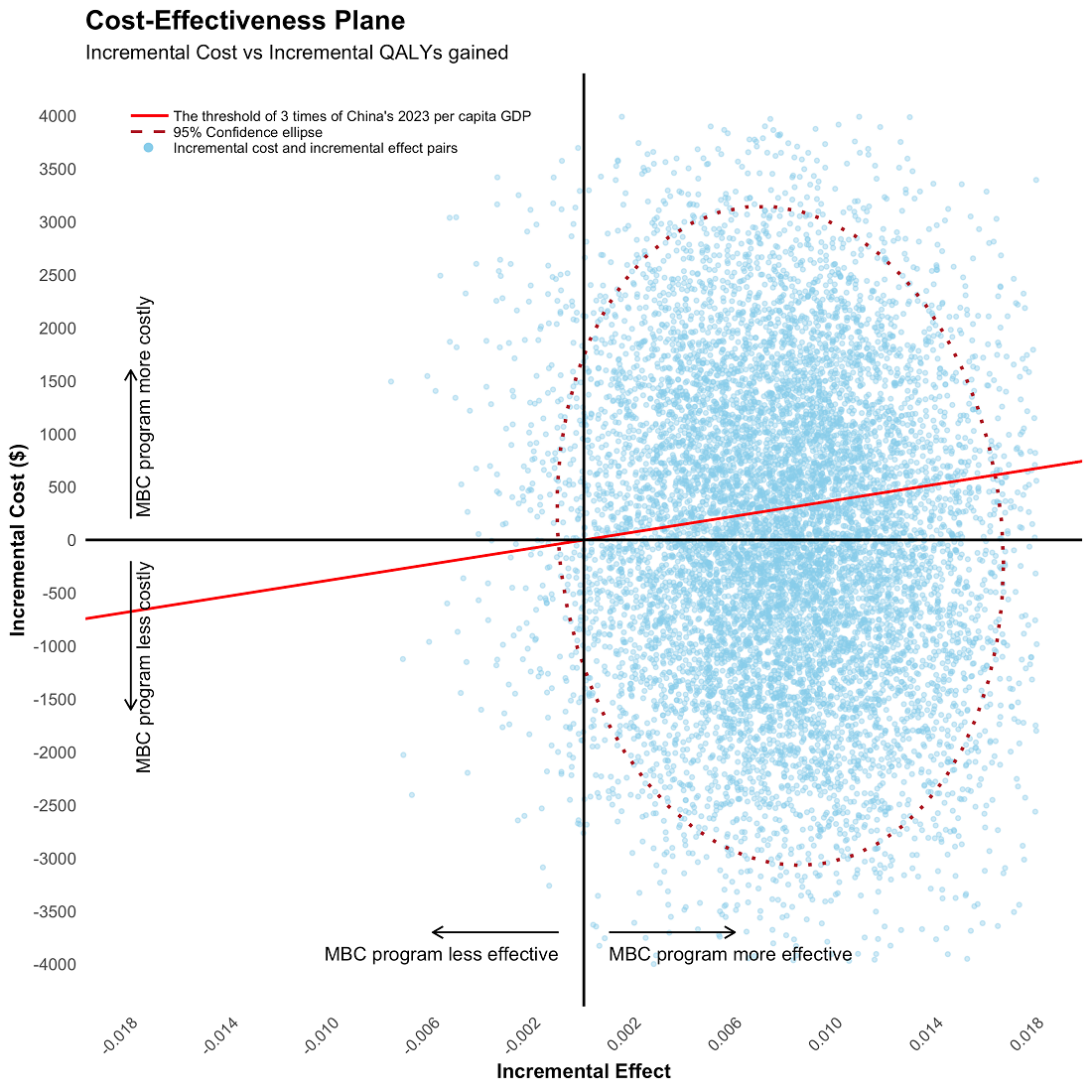
Cost-effectiveness plane of QALYs gained comparing intervention group to control group at T2. QALYs: quality-adjusted life years; MBC: mindfulness breast care.

**Figure 6:**
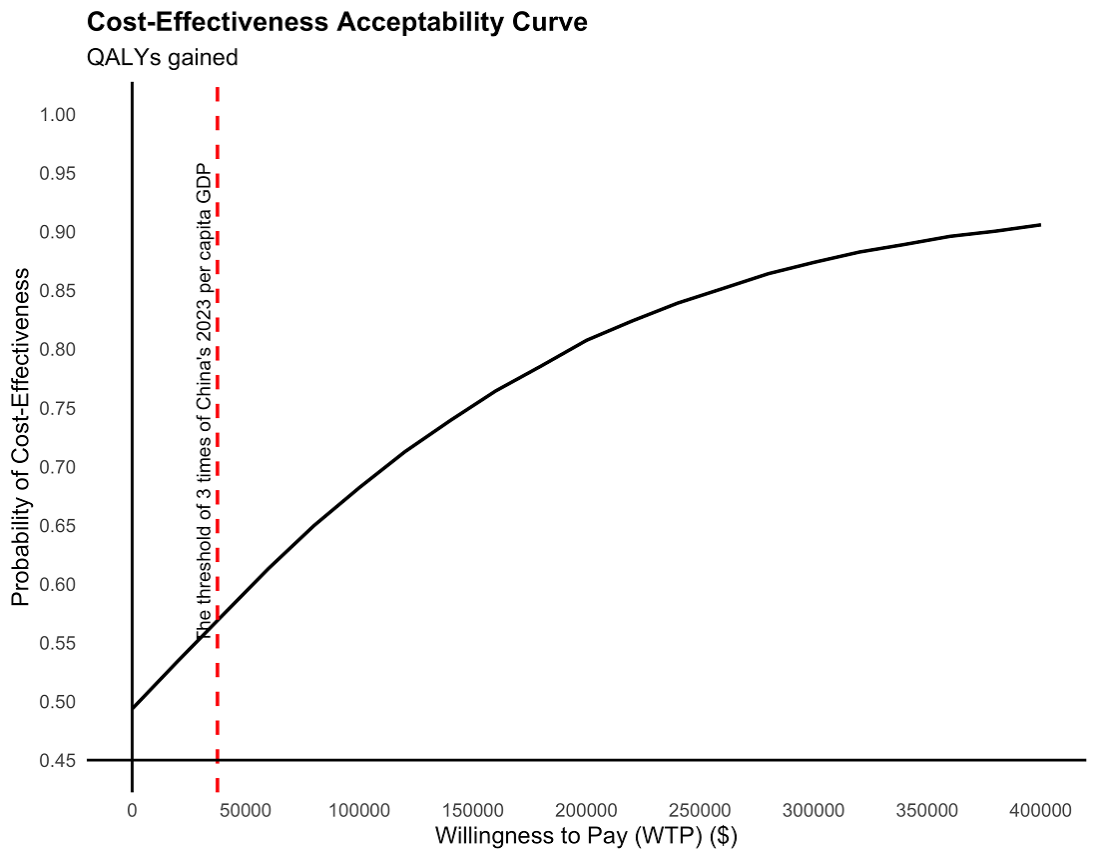
Cost-effectiveness acceptability curve of QALYs gained comparing intervention group to control group at T2. QALYs: quality-adjusted life years.

The robustness of this finding was confirmed by the sensitivity analyses. The cost-effectiveness plane showed the ICERs of QALYs gained were predominantly located in the southeast and northeast quadrants, indicating that the cost-effectiveness of these outcomes may be dominant or can be evaluated by comparison with the threshold (US $37,530; Figure 5). The cost-effectiveness acceptability curve predicted that our program had a 57% probability of being cost-effective in QALYs gained (Figure 6).

### Usage Data and Its Relationships With Measurement Outcomes

The 3-month usage data of MBC program varied considerably (Table 5). The median of the total usage duration (minutes) was 199.60 (IQR 70.90-451.31, mean 360.59, SD 511.72), and the total login frequency (time) was 39.50 (IQR 19.00-86.50, mean 57.02, SD 50.26). In our intervention, a total of 76 participants (76/96, 79.2%) met the expected use as specified beforehand. The Mindfulness Practices module was most popular among participants (median usage duration 124.06, IQR 8.45-330.33; login frequency 22, IQR 8.25-43.50), followed by the Library (median usage duration 25.54, IQR 5.82-111.75; login frequency 11, IQR 4-26) and Mindfulness Yoga modules (median usage duration 3.89, IQR 0.12-16.91; login frequency 2, IQR 1-6) (Table 5). No moderate or strong correlation between MBC usage data and measurement outcomes was found.

**Table 5:** Usage data of the Mindfulness Breast Care program.

Program and modules	Usage duration (minutes)				Login frequency (times)		
	Median (IQR)	Mean (SD)	Maximum	Median (IQR)		Mean (SD)	Maximum
						
Entire MBC^a^ program	199.60 (70.90-451.31)	360.59 (511.72)	3382.82	39.50 (19-86.50)		57.02 (50.26)	239
Library	25.54 (5.82-111.75)	144.32 (427.31)	3177.62	11 (4-26)		22.05 (28.34)	142
Mindfulness yoga	3.89 (0.12-16.91)	17.97 (32.37)	160.58	2 (1-6)		4.81 (7.58)	57
Mindfulness practices	124.06 (8.45-330.33)	198.31 (218.73)	1088.80	22 (8.25-43.50)		30.16 (28.27)	128

^a^MBC: Mindfulness Breast Care.

## Discussion

### Principal Results

This is the first RCT to investigate the effectiveness and cost-effectiveness of an app-based program for survivors of breast cancer in China. Our study illustrates that MBC program can decrease body image distress and stigma, and improve mental well-being for survivors of breast cancer. Our study also demonstrates the potential cost-effectiveness of our program.

Our program statistically significantly reduced body image distress at six months, not at three months (post intervention). Prior study shows that a weekly 90-minute mindfulness-based meditation and yoga intervention significantly reduced body image distress for patients with breast cancer immediately post intervention [72]. Our study only offered weekly 45-minute videoconference on mindfulness practice and may not be sufficient to achieve optimal effects immediately. However, survivors of breast cancer can gradually enhance their mindfulness and yoga skills through cumulative practice, thereby better observing and accepting body changes at follow-up [73]. Meanwhile, the trial did not show a clinically meaningful difference of body image distress. A systematic review recommends combining psychological interventions with physical activities and cosmetic procedures for better results [74].

Our program statistically significantly decreased stigma at three and six months, which was consistent with the results from two prior studies demonstrating that face-to-face MBCT theory–driven intervention significantly decreased stigma of patients with schizophrenia [25,75]. Additionally, an important clinical difference at three and six months was discovered, indicating that the impact of MBC program on stigma was clinically meaningful. Mindfulness practices and yoga could promote awareness of negative thoughts among survivors of breast cancer, such as stigma, and treat them in a non-judgmental manner, ultimately leading to stigma reduction [12]. Notably, statistically significant time effects, as well as time and group interaction effects regarding stigma were also found, which indicated that the impact of our program was sustained and might be endured over time.

In contrast to the prior 2 studies [76,77], our program did not statistically significantly improve sleep quality. The difference between our study and the prior two studies may be explained by the density and format of interventions, with weekly 45-minute video conferencing sessions in our intervention and weekly two-hour face-to-face group sessions in the prior two interventions [76,77]. Longer immersion in mindfulness practices and face-to-face communication may enhance the impact of mindfulness on sleep quality.

Our program improved mental well-being for participating survivors of breast cancer at three months but not sustained at six months. Mindfulness practices may be helpful for participants to regulate feelings consciously, deal with emotions peacefully, and improve their mental stability [78,79]. However, such effects would not be sustained when participants felt unsupported after the intervention was completed [80]. Hence, more scientific researches are encouraged to extend the duration of app-based MBCT-guided programs for reaching longer-term mental well-being.

Time effect on social support was found at the 6-month follow-up. Although the group difference in social support at T2 did not reach statistical significance (*P*=.06), this study suggested a trend toward improvement. Mindfulness and stigma reduction may facilitate BCSs to build stronger relationships with others, thus promoting their social support [81].

Participating survivors of breast cancer reported better QALYs in our program. A systematic review found that all app-based programs were cost-effective and reported greater QALYs gained compared to routine care [82]. Given the stretched resources in health care provision [83] and the continuous rise in the number of cancer survivors [1], an easily accessible, sustainable, clinically effective, and relatively cost-effective strategy for posttreatment care is much needed [84,85]. Our study provides further evidence for health care budget holders when considering continuity of care after discharge.

In our study, the probability that the MBC program is cost‑effective was 57% at a willingness‑to‑pay threshold of US $37,530 per QALY, which is in line with the concept of “more value for money” [86]. However, the cost‑effectiveness probability of 57% reflects the inherent uncertainty of trial‑based economic evaluations, particularly given the modest sample size and short analytic horizon [52]. Prior methodological literature indicates that probabilities of cost-effectiveness around 50–60% are common in exploratory or early‑phase studies, and should be interpreted as decision uncertainty rather than evidence against cost‑effectiveness [87,88]. Furthermore, owing to the lack of a guideline on the perceived acceptable probability of cost-effectiveness of internet programs by health care policymakers [89], future economic evaluations of app-based programs are needed to help draw a firm conclusion.

In our 12-week program, the average login frequency and usage duration of the entire program were 57.02 times and 360.59 minutes, respectively. This means that within the 12-week intervention period, women logged into MBC program approximately 4-5 times per week and used the program 30 minutes per week. This intervention showed better usage data than previous studies [55,90]. A prior 16-week web-based program for BCSs indicated the average of total login frequency and usage duration as 11 times and 337.2 minutes [55], and another 24-week app-based program for patients with gynecologic cancer reported the average of total login frequency to be 67.90 times and usage duration to be 85.23 minutes [90]. The better usage data in our program could be partially explained by the weekly 45-minute videoconference, where all participants were invited to practice mindfulness with the therapist during the videoconference. Participants’ interactivity positively influenced their engagement in an app-based intervention [91].

In our program, the relatively large difference between mean and median revealed usage polarization among survivors of breast cancer. Usage polarization of the MBC program could be due to the variation in participants’ education level, with 38.5% of participants’ educational level at junior high school or lower. Participants with less educated might not be familiar with novel technology, resulting in less use [92]. Future researchers should consider offering more instruction on app usage with plain language and applying various strategies to make app-based programs more understandable and acceptable.

### Limitations

Certain limitations warrant consideration. First, the sample size was calculated exclusively on the effectiveness parameter while neglecting the economic parameter (ie, QALYs), rendering our study unlikely to be adequately robust for an economic analysis [93]. Second, we did not incorporate a face-to-face mindfulness intervention into our design, making it impossible to clarify the differences in costs and effects between different formats (app-based or face-to-face) of MBCT-guided intervention. However, internet-based interventions offer a potential platform for one mindfulness therapist to serve more cancer survivors, as well as save participants’ transportation costs and time to achieve cost savings [27]. Third, owing to limited resources, only the cost-benefit within 6 months was evaluated, and the long-term effects were not considered. A short period may not fully demonstrate their cost-effectiveness, especially for health care interventions with high upfront costs and potential long-term effects [94]. Fourth, data collectors and participants were not blinded to group allocation, which may increase the risk of unconscious and conscious bias during data collection in an RCT. Further, self-report outcomes may be subject to reporting bias [95].

### Conclusions

Our trial reveals that an app-based MBC program is effective and potentially cost-effective. Our study indicates that offering this program in clinical practice reduces body image distress and stigma, improves mental well-being, and saves societal costs. It is recommended that this program be incorporated into routine care to support cancer patients after primary treatment. Health care policymakers should consider allocating more resources to eHealth.

## Data Availability

All data collected have been shared in Mendeley Data (DOI: 10.17632/7hsp3jnwh8.1). All data analysis results of this study are included in this article.

## Appendix

Multimedia Appendix 1 CONSORT-eHEALTH checklist (V 1.6.1).

Multimedia Appendix 2 CHEERS 2022 checklist.

## Abbreviations

BIS: Body Image Scale
CHEERS: Consolidated Health Economic Evaluation Reporting Standards
GDP: gross domestic product
ICER: incremental cost-effectiveness ratio
MBC: mindfulness breast care
MBCT: mindfulness-based cognitive therapy
MCS: Mental Component Summary
MSPSS: Multidimensional Scale of Perceived Social Support
PCS: Physical Component Summary
PSQI: Pittsburgh Sleep Quality Index
QALY: quality-adjusted life year
QoL: quality of life
RCT: randomized controlled trial
SF-6D: Short-Form Six-Dimension
SF-36: 36-item Short-Form Health Survey
SIS: Social Impact Scale
