# Supplementary material for: Effectiveness and Cost-Effectiveness of an App-Based Mindfulness Breast Care Program for Reducing Body Image Distress and Stigma Among Survivors of Breast Cancer: Randomized Controlled Trial
Source: J Med Internet Res. 2026 Apr 24;28:e85913. doi: 10.2196/85913 (PMC13156539; doi:10.2196/85913)
Supplement: Multimedia Appendix 2 [file jmir_v28i1e85913_app2.pdf]

ISPOR
Improving healthcare decisions

Consolidated Health Economic Evaluation Reporting Standards (CHEERS) 2022 Checklist

The CHEERS 2022 statement replaces the 2013 CHEERS statement, which should no longer be used. The CHEERS 2022 checklist contains 28 items with accompanying descriptions. Checklist users should indicate the section of the manuscript where relevant information can be found. The authors recommend using a section heading with a paragraph number. If an item does not apply to a particular economic evaluation, checklist users are encouraged to report "Not Applicable." If information is otherwise not reported, checklist users are encouraged to write, "Not Reported." Users should avoid the term "Not Conducted" as CHEERS is intended to guide and capture reporting. Additional information on CHEERS 2022 can be found here.

Title

1. Title

Identify the study as an economic evaluation and specify the interventions being compared.

Effectiveness and cost-effectiveness of an app-based mindfulness breast care program to reduce body image distress and stigma for breast cancer survivors: Randomized controlled trial

---

Abstract

2. Abstract

Provide a structured summary that highlights context, key methods, results, and alternative analyses.

Background: Breast cancer surgery and corresponding treatments have significant residual effects on breast cancer survivors (BCSs) in China. Body image distress and stigma are persistent challenges that negatively affect their quality of life. Accessible, sustainable, and cost-effective support remains scarce.
Objective: This trial aimed to evaluate the effectiveness and cost-effectiveness of an app-based mindfulness breast care (MBC) program in addressing body image distress and stigma for BCSs.
Methods: A randomized controlled trial was carried out in 2 university-affiliated hospitals in China. BCSs who had completed primary treatments and had mobile phone internet access were recruited and randomly assigned at 1:1 ratio to the intervention (3-month MBC program plus routine care) or the control group (routine care alone). Under the conceptualization of Mindfulness-based cognitive therapy, the MBC program was developed including 3 modules: (1) Library, (2) Mindfulness Yoga, and (3) Mindfulness Practices. The primary outcomes measured were body image distress and stigma, and secondary outcomes included sleep quality, social support, and quality of life (physical and mental well-being). Assessments were conducted at baseline, 3

months (T1), and 6 months (T2). An intention-to-treat approach, utilizing a multiple imputation method, was employed to handle missing data. Generalized estimating equations were fitted to evaluate the effectiveness. The incremental cost per quality-adjusted life year (QALY) gained was used to measure the cost-effectiveness.
Results: A total of 192 BCSs participated in the baseline assessment, with 155 completing the two follow-up surveys. The median of the total usage duration (minute) was 199.60 (IQR 70.90-451.31, mean 360.59, SD 511.72), and total login frequency (time) was 39.50 (IQR 19.00-86.50, mean 59.26, SD 50.26). The reduction in body image distress at T2 (adjusted mean difference, −1.91; 95% CI, −3.40 to −0.42, P=.012, d=−0.31), the reduction in stigma at T1 (adjusted mean difference, −5.83; 95% CI, −8.46 to −3.20, P<.001, d=−0.61) and T2 (adjusted mean difference, −7.79; 95% CI, −10.62 to −4.97 P<.001, d=−0.82), and the improvement in mental well-being at T1 (adjusted mean difference, 4.44; 95% CI, 1.70 to 7.18, P=.002, d=0.43) were statistically significantly greater in the intervention group compared to the control group. No statistically significant group differences were observed regarding sleep quality, social support, and physical well-being. The cost-effectiveness analysis showed that the intervention group gained more QALYs than the control group at T2 (adjusted mean difference, 0.008; 95% CI, 0.004 to 0.016, P=.012). The incremental cost per QALY gained at T2 was $19,431.25, indicating a 57.00% probability that MBC program is a cost-effective intervention at a threshold of $37,530, three times of China's 2023 GDP per capita.
Conclusions: An app-based mindfulness breast care program was effective and potentially cost-effective, and had the promise to be scalable for clinical practice.

Introduction

3. Introduction: Background and Objectives

Give the context for the study, the study question, and its practical relevance for decision making in policy or practice.

Details can be seen in Introduction section.
The context for the study: Few app-based studies included health economic evaluation.
The study question: It aims to evaluate the cost-effectiveness of an app-based Mindfulness Breast Care (MBC) program.
The practical relevance for decision making in practice: The results gained from the trial will provide evidence for health policymakers and hospitals to allocate more resources and train staff for e- health.

Methods

4. Health economic analysis plan

Indicate whether a health economic analysis plan was developed and where available.

A health economic analysis plan was developed and was described in detail in Economic evaluation measures and Cost-effectiveness of Method section.

5. Study population

Describe characteristics of the study population (such as age range, demographics, socioeconomic, or clinical characteristics).

Eligible participants were women with breast cancer who have completed primary treatments and were ready to be discharged. Details in Study design and participants section, the 2nd paragraph.

6. Setting and location

Provide relevant contextual information that may influence findings.

There were no other app-based programs to support BCSs at the two participating hospitals during the study period. Details in Study design and participants section, the 3rd paragraph.

7. Comparators

Describe the interventions or strategies being compared and why chosen.

Participants in two groups received routine care. Before discharge, nurses offer oral and written instructions on routine post-cancer treatment information and follow-up education. At every follow-up visit, oncologists conduct medical assessments and adjust medications as needed. Nurses give general advices on monitoring and managing symptoms post-treatments. Details in Routine Care section.

8. Perspective

State the perspective(s) adopted by the study and why chosen.

An economic evaluation was conducted alongside the trial to explore the cost-effectiveness of the intervention group compared with the control group from societal perspective, expressing in term of additional cost per Quality Adjusted Life Years (QALYs) gained.

9. Time horizon

State the time horizon for the study and why appropriate.

A time horizon of 6-month was made, namely, the time duration of the trial and follow-up. Breast cancer survivors at the two participating hospitals were often followed up once per 3 months.

---

10. Discount rate

Report the discount rate(s) and reason chosen.

Assuming the app remains operational for 5 years, fixed costs associated with the MBC programme were calculated using a depreciation factor of 3% for consultation fees. because the time duration of the trial was within a year, the effectiveness outcomes were not discouted. Details in Cost-effectiveness of Methods section, the 2nd paragraph.

---

11. Selection of outcomes

Describe what outcomes were used as the measure(s) of benefit(s) and harm(s).

Total costs and QALYs gained were used to calculate the Incremental Cost-Effectiveness Ratio (ICER) and net monetary benefit statistics.

---

12. Measurement of outcomes

Describe how outcomes used to capture benefit(s) and harm(s) were measured.

According to the recommendation of World Health Organisation (WHO), three times of China's 2023 per capita GDP ($12,510) was applied as the cost-effectiveness threshold. The interpretation was as follows: If ICER < 3 times GDP ($37,530), it is deemed to have a cost-effectiveness value.

---

13. Valuation of outcomes

Describe the population and methods used to measure and value outcomes.

Details in Cost-effectiveness of Methods section, the 4th paragraph. populations included the trial participants. study used cost-effectiveness analysis to measure outcomes, expressing in terms of ICER-QALYs gained.

---

14. Measurement and valuation of resources and costs

Describe how costs were valued.

© 2022 ISPOR — The Professional Society for Health Economics and Outcomes Research

International Society for Pharmacoeconomics and Outcomes Research, Inc

Details in Cost-effectiveness of Methods section, the 1st -3rd paragraph.
The costs mentioned in this study included three components: (1) fixed costs associated with the app development; (2) direct medical costs; and (3) indirect costs, namely income and time lost incurred due to medical treatments.

---

15. Currency, price date, and conversion

Report the dates of the estimated resource quantities and unit costs, plus the currency and year of conversion.

Data was collected as the CNY currency unit. Study reported USD as the currency unit. All monetary values were converted to 2023 USD using the 2022/2023 average conversion rate.

---

16. Rationale and description of model

If modeling is used, describe in detail and why used. Report if the model is publicly available and where it can be accessed.

We did not use a model.

---

17. Analytics and assumptions

Describe any methods for analyzing or statistically transforming data, any extrapolation methods, and approaches for validating any model used.

Details in Cost-effectiveness of Methods section, the 5th paragraph.

---

18. Characterizing heterogeneity

Describe any methods used for estimating how the results of the study vary for subgroups.

We did not perform the subgroups analysis.

---

19. Characterizing distributional effects

Describe how impacts are distributed across different individuals or adjustments made to reflect priority populations.

Details in Cost-effectiveness of Methods section, the 4th paragraph.
GEEs were fitted to evaluate the direct and indirect costs (adjusted for endocrine therapy) and the QALYs gained (adjusted for endocrine therapy and baseline health utility scores).

20. Characterizing uncertainty

Describe methods to characterize any sources of uncertainty in the analysis.

Details in Cost-effectiveness of Methods section, the 5th paragraph.
These 10,000 bootstrap pairs of incremental costs and QALYs were graphically represented on the cost-effectiveness plane to illustrate the uncertainty surrounding ICER at the threshold above mentioned.

21. Approach to engagement with patients and others affected by the study

Describe any approaches to engage patients or service recipients, the general public, communities, or stakeholders (eg, clinicians or payers) in the design of the study.

An instant messaging reminder was designed in our app-based program. Participants received an appreciation gift of approximately $3 after each evaluation.

Results

22. Study parameters

Report all analytic inputs (eg, values, ranges, references) including uncertainty or distributional assumptions.

Parameter inputs were data of the trial adjusted by confunding covariates. This was described in detail in Cost-effectiveness section.

23. Summary of main results

Report the mean values for the main categories of costs and outcomes of interest and summarize them in the most appropriate overall measure.

Details in the cost-effectiveness of results section and Table 4.
The total costs were estimated at $9,295.45 for the intervention group and $9,140.00 for the control group with no statistically significant group difference (P=.917). The intervention group gained more quality-adjusted life years (QALYs) than the control group at T2 (adjusted mean difference 0.008, 95% CI, 0.004 to 0.016, P=.012). The incremental cost per QALY gained at T2 was $19,431.25, making our program cost-effective at a threshold of three times of China's 2023 GDP per capita ($37,530) (Table 4).

24. Effect of uncertainty

Describe how uncertainty about analytic judgments, inputs, or projections affects findings. Report the effect of choice of discount rate and time horizon, if applicable.

Details in the cost-effectiveness of results section(last paragraph), discussion and limitations section.
Our sensitivity analysis reported a 57% probability that MBC program was a potential cost-effective intervention at a willingness-to-pay threshold. Our result illustrates that participants who used MBC program gained more QALYs, and they were willing to pay for it, which is in line with the concept of "more value for money" .
However, the cost-effectiveness probability of 57% reflects the inherent uncertainty of trial-based economic evaluations, particularly given the modest sample size and short analytic horizon . Prior methodological literature indicates that probabilities of cost-effectiveness around 50–60% are common in exploratory or early-phase studies, and should be interpreted as decision uncertainty rather than evidence against cost-effectiveness . Furthermore, owing to the lack of a guideline on the perceived acceptable probability of cost-effectiveness of internet programs by healthcare policymakers , future economic evaluations of app-based programs are needed to help draw a firm conclusion.
First, the sample size was calculated exclusively on the effectiveness parameter while neglecting the economic parameter (i.e. QALYs), rendering our study unlikely to be adequately robust for an economic analysis [93].

---

25. Effect of engagement with patients and others affected by the study

Report on any difference patient/service recipient, general public, community, or stakeholder involvement made to the approach or findings of the study.

This was described in detail in effectiveness of Result section.

Discussion

26. Study findings, limitations, generalizability, and current knowledge

Report key findings, limitations, ethical, or equity considerations not captured and how these could impact patients, policy, or practice.

Our multicentre RCT contributes to the evidence that the app-based MBC program is effective and potential cost-effective. Certain limitations warrant consideration. First, the sample size was calculated exclusively on the effectiveness parameter while neglecting the economic parameter (i.e. QALYs), rendering our study unlikely to be adequately robust for an economic analysis. Second, we did not incorporate a face-to-face mindfulness intervention into our design, making it impossible to clarify the differences in costs and effects between

different formats (app-based or face-to-face) of MBCT-guided intervention. Third, owing to limited resources, only the cost-benefit within six months was evaluated, and the long-term effects were not considered. A short period may not fully demonstrate their cost-effectiveness, especially for healthcare interventions with large upfront costs and potential long-term effects.

Other Relevant Information

27. Source of funding

Describe how the study was funded and any role of the funder in the identification, design, conduct, and reporting of the analysis.

We received support from the National Natural Science Foundation of China (71974162 and 7231101009). The funding bodies provided the financial support for the MBC development and clinical implementation. The funding bodies had no role in the design of the study, data collection, analysis, and interpretation, the manuscript writing, or submission decision.

---

28. Conflicts of interest

Report authors' conflicts of interest according to journal or International Committee of Medical Journal Editors requirements.

All authors declare no conflicts of interest.
